# Enhancing thermal stability of Nd:GGG WGM microdisk lasers via silica integration

**DOI:** 10.1515/nanoph-2024-0011

**Published:** 2024-04-15

**Authors:** Huiqi Li, Zhaocong Wang, Lei Wang, Yang Tan, Feng Chen

**Affiliations:** School of Physics, State Key Laboratory of Crystal Materials, 12589 Shandong University, Jinan 250100, China

**Keywords:** whisper gallery mode, ion implantation, crystalline film, microlaser

## Abstract

Whispering gallery mode (WGM) resonators, as an integral component of integrated photonics, have attracted considerable attention due to their high *Q* factor, small footprint, and small mode volume, making them widely applied as microlasers. In this work, Nd:GGG crystal was prepared into a Nd:GGG film with thickness of 1.8 μm through ion implantation-enhanced etching (IIEE) technique, and subsequently, the Nd:GGG film was partened by focused ion beam (FIB) technology to generate a microdisk with diameter of 20 μm. For high-power microcavity lasers, heat generation during laser operation was inevitable. We placed the microdisk on a silica holder and a silica wafer, respectively. The microdisk placed on the silica holder and silica wafer exhibited laser thresholds of 32 μW and 17 μW, respectively. Moreover, due to different heat dissipation conditions, the microdisk placed on the silica holder exhibited a mode shift of 0.13 nm/mW, while the microdisk placed on the silica wafer showed a more stable laser output state with a mode shift of 0.02626 nm/mW.

## 1 Introduction

WGM resonators, which confine light through total internal reflection in a small volume, have found widespread applications in various fields such as Raman enhancement [1], [2], [3], nonlinear optics [4], [5], [6], quantum communication [7], [8], high-sensitivity sensors [9], [10], [11], gain lifetime characterization [12], and on-chip microlasers [13], [14], [15], [16]. In particular, WGM resonators with small mode volumes are highly desirable for on-chip cavity lasers including both electrically [17] and optically pumped [18], as they enable ultra-low laser thresholds [19] and narrow linewidth laser output [20]. For optically pumped WGM lasers, the laser performance depends on the *Q* factor of the WGM cavity and the properties of the gain medium. Silica and lithium niobate (LNOI) are two materials commonly used for WGM resonators, with high *Q* factors. Rare-earth-doped silica is advantageous due to its low melting point, which allows for the realization of resonators with ultra-high *Q* factors. This is achieved by exposing the microdisk to a CO_2_ laser beam, resulting in surface tension [21], [22]. This makes it suitable for sensor [23], [24], [25] and non-Hermitian optics [26], [27]. On the other hand, rare-earth-doped LNOI possesses excellent photoelectronic and nonliner properties, making it an ideal medium for ultrafast integrated optics. However, while these WGM resonators have ultra-high *Q* factors, the rare-earth-doped silica and LNOI are not exceptional gain media. As a result, WGM lasers based on these materials have low laser output power (not exceeding 10 μW) and low optical conversion efficiency (lower than 1.5 %) [28], [29], [30]. Therefore, selecting an exceptional gain medium for WGM lasers is crucial for achieving high laser power and optical conversion efficiency.

Rare-earth-doped garnet crystals, such as yttrium aluminum garnet (YAG), possess exceptional lasing properties such as high absorption and emission cross sections, making it an ideal choice as gain medium for solid state lasers in commercial applications [31], [32], [33]. Recently, rare-earth-doped YAG crystals have been utilized in the fabrication of microdisk lasers, resulting in laser output of 1 mW with an optical conversion exceeding 10 % [34], [35]. However, the low refractive index of YAG crystal (∼1.83) necessitates suspending the YAG microdisk boundary in air to increase the confinement capability of the resonance modes. Unfortunately, air has poor thermal conductivity, leading to heat accumulation within the microdisk during laser operation. This adversely affects laser performance by causing a redshift in the laser frequency due to optothermal effect and thermal expansion of the rare-earth-doped YAG microdisk. To effectively address this issue, placing the microdisk on a high thermal conductivity substrate helps to lower its temperature. However, this also reduces the confinement capability of the resonance modes in YAG microdisk. It is necessary to choose a material with a higher refractive index for the microdisk to achieve effective mode confinement and efficient heat dissipation, enabling the stable emission of microdisk lasers.

Gadolinium gallium garnet (GGG), as a member of the garnet crystal family, has a higher refractive index (∼1.94) compared to YAG, meeting the requirements for combining microdisks with high thermal conductivity substrates. Additionally, Nd:GGG allows for a higher doping concentration of 4 % due to the weak quenching of Nd^3+^ ions in GGG, whereas in YAG, the doping concentration is limited to 1.5 % [36]. This makes Nd:GGG an excellent laser medium for high-power solid-state lasers operating at 1.06 μm and 1.3 μm [37], [38], [39]. Therefore, utilizing rare-earth-doped GGG crystals as the gain medium in microdisk lasers shows great potential for enhancing thermal stability. Due to the high transmittance of near-infrared lasers in the atmosphere [40], they have significant practical value in areas such as laser radar and environmental monitoring. Furthermore, the unique biophysical characteristics of this band make it widely applicable in the field of biomedical [41]. This creates favorable conditions for the application of Nd:GGG microdisks in fields such as environment and biomedicine.

Here, we have utilized Nd:GGG crystal to fabricate microdisk integrated with a silica wafer, resulting in minimal redshift of the lasing mode. To prepare the Nd:GGG microdisks, we have employed an IIEE method to separate a 1.8 μm thick crystalline film from the Nd:GGG crystal. The Nd:GGG film has been milled into a microdisk with a diameter of 20 μm using the FIB milling. To evaluate the impact of heat dissipation on laser performance, the Nd:GGG microdisk was carefully positioned on a silica holder and a silica wafer, respectively, allowing for a comparative analysis. Under 808 nm laser pumping, the Nd:GGG microdisk exhibits a redshift rate (a laser threshold) of 0.13 nm/mW (32 μW) when positioned on the silica holder, whereas when directly placed on a silica wafer, the redshift rate (laser threshold) decreases to 0.02626 nm/mW (17 μW), indicating superior thermal stability of the microdisk integrated with silica wafer compared to the one on the silica holder. This improvement can be attributed to the high thermal conductivity of silica, which enhances heat dissipation of the Nd:GGG microdisk. This work demonstrates the exceptional potential of rare-earth doped GGG crystals as gain materials for microdisk lasers, enabling them to achieve thermal stability and integration.

## 2 Preparation of Nd:GGG microdisk

**Figure 1:**
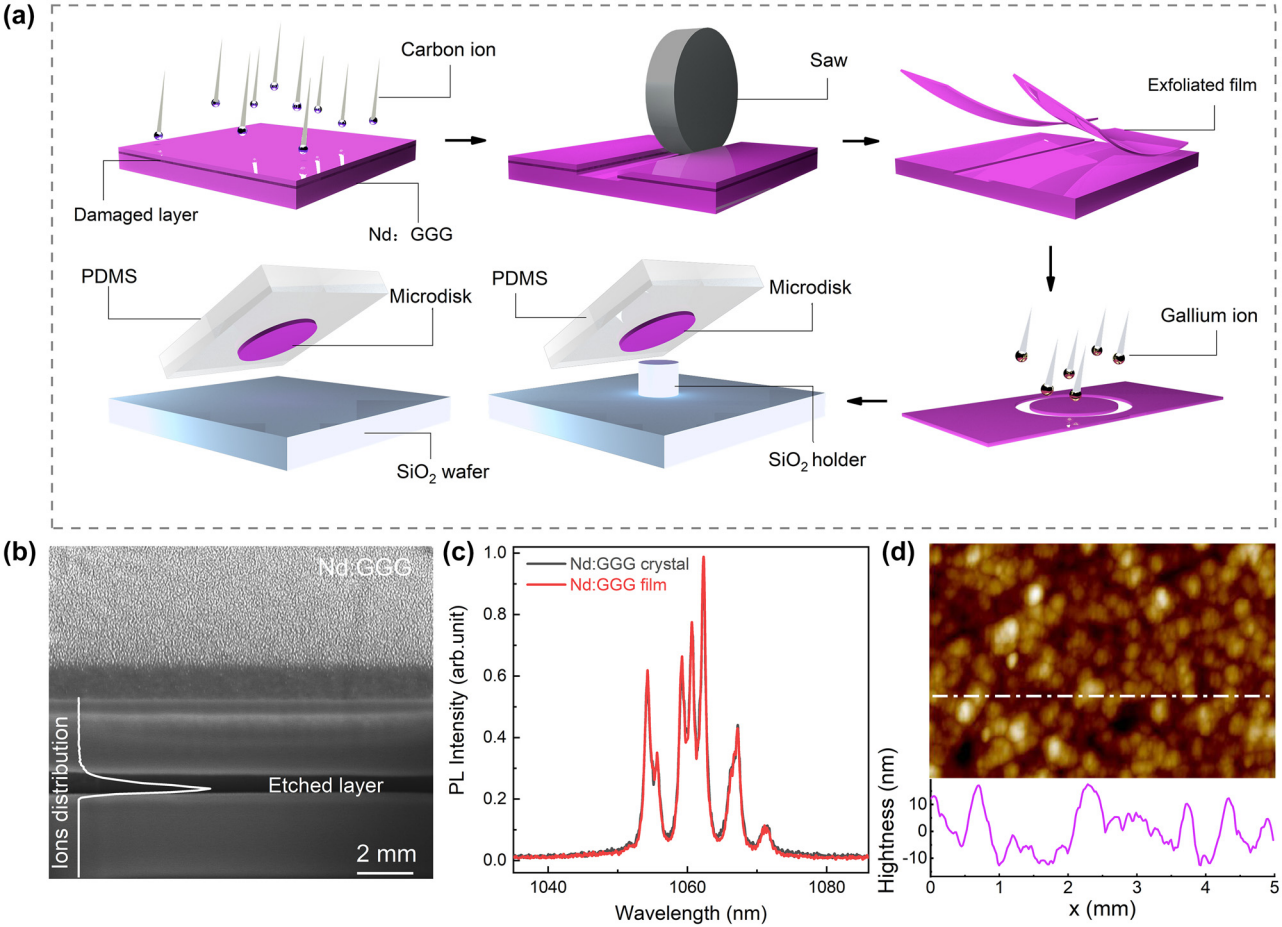
Preparation of Nd: GGG microdisk. (a) Schematic diagram of the fabrication of Nd:GGG crystal thin film and microdisk; (b) SEM image of the Nd:GGG after etched. Scale bar, 2 μm; (c) normalized PL spectra of Nd:GGG crystal (black line) and Nd:GGG film (red line) ion implantation; (d) AFM image of exfoliated Nd:GGG film.

Figure 1a illustrates the fabrication process of the Nd:GGG microdisk. A Nd:GGG film is exfoliated by the IIEE method. The Nd:GGG crystal utilized in this study had dimensions of 10 × 10 × 1 mm^3^, with the largest facets (10 × 10 mm^2^) optically polished. One of the largest facets was implanted with carbon ions at a dose of 3 × 10^15^ ions/cm^2^ and an energy of 5 MeV, at an angle of 7° away from the surface normal. The incident carbon ions lose energy through collisions with atoms in Nd:GGG crystal, ultimately coming to rest inside the crystal and forming a damaged layer. Next, multiple parallel grooves were created on the implantation surface using a diamond scribe (with a spacing of 100 μm) to ensure the exposure of the damaged layer. The depth of the grooves is approximately 10 μm. After annealing, the Nd:GGG crystal was immersed in a phosphoric acid solution at 70 °C for 30 min to etch the damaged layer, thereby separating the Nd:GGG surface film from the crystal and obtaining an Nd:GGG crystalline film with a thickness of approximately 1.8 μm. To observe the etching process clearly, a slice of the Nd:GGG crystal was taken out during the etching process. The residual phosphoric acid on the crystal was washed off, and the etching cross-sections are characterized using scanning electron microscopy (SEM) in Figure 1b. It can be observed that the damaged layer is etched, and the Nd:GGG crystal above and below the etched layer are well preserved.

The Nd:GGG crystalline thin film was transferred to a silica substrate using Polydimethylsiloxane (PDMS). And the crystalline thin film was patterned using FIB milling to obtain Nd:GGG microdisk with a diameter of 20 μm and a thickness of 1.8 μm. Subsequently, the Nd:GGG microdisk was selectively picked up from the substrate using PDMS and aligned with the target substrate with the assistance of an inverted microscope. Meanwhile, we used a diamond scribe to prepare a silica holder. Initially, a groove was cut approximately 8 μm away from the edge. Subsequently, the substrate was rotated by 90°, and the same procedure was repeated on the adjacent side to obtain a rectangular silica holder. Depending on the requirements, the Nd:GGG microdisk was either placed on a pre-fabricated silica holder or directly positioned onto a clean silica wafer.

Figure 1c shows the photoluminescence (PL) spectrum of the Nd:GGG crystal (black solid line) and Nd:GGG film (red line). The PL peak positions and intensities are similar for both cases, indicating the IIEE process has no significant effect on the PL properties of the Nd:GGG crystal. The roughness of the exfoliated crystalline film was characterized by atomic force microscopy (AFM) (Figure 1d), and no obvious etching pits are observed on the surface of the film, with a roughness of 8.38 nm rms.

**Figure 2:**
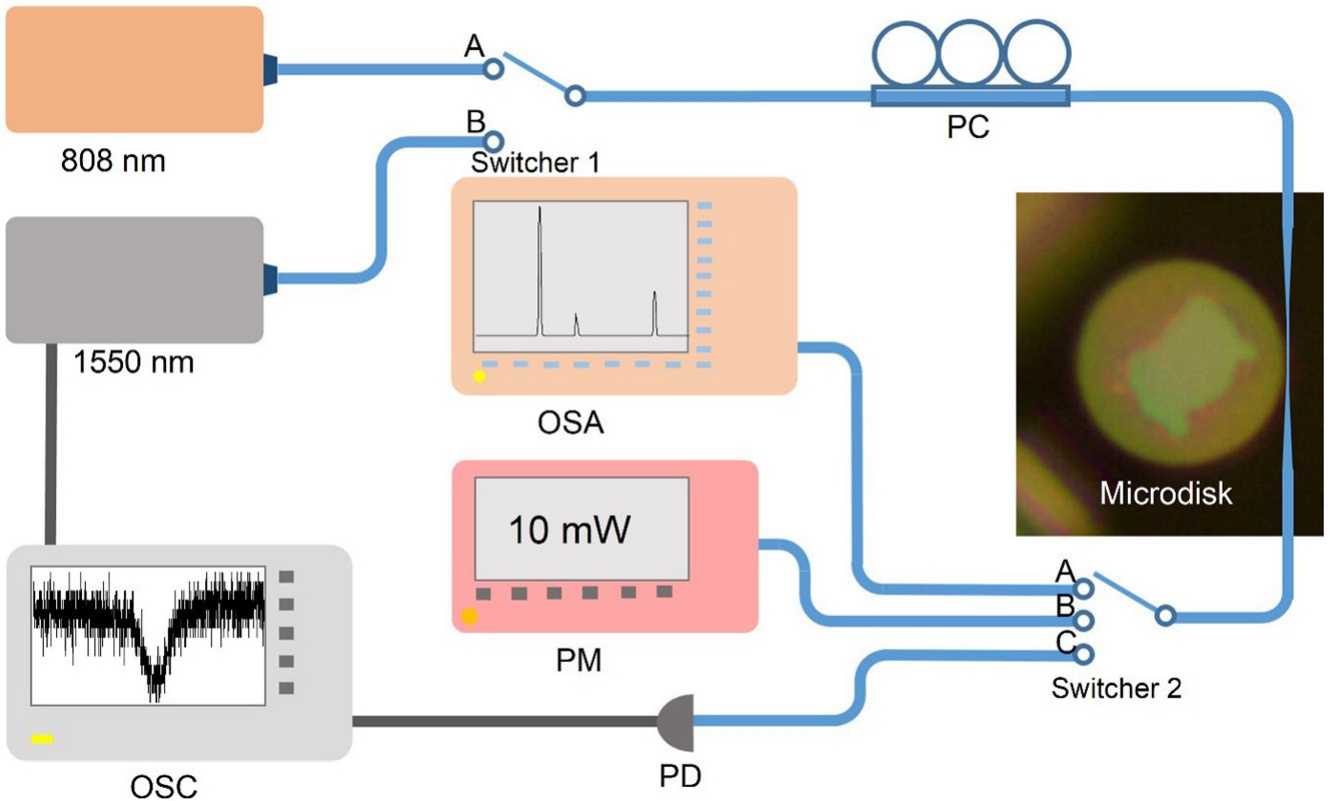
Experimental setup for Nd:GGG microdisk laser. PC, polarization controller; OSA, optical spectrum analyzer; PM, power meter; PD, photodetector; OSC, oscilloscope.

To characterize the optical properties of the Nd:GGG microdisk, we conducted transmission spectrum measurements using the experimental setup depicted in Figure 2, with switcher 1 set to position B and switcher 2 also set to position C. In this setup, a tunable laser serves as the probe light to detect the transmission spectrum. The wavelength of the probe light is carefully selected within the range of 1500–1540 nm to avoid any optical absorption induced by the doped Nd ions. The polarization state of the probe light is adjusted using a polarization controller (PC) and then guided into a tapered fiber. When the probe light is directly connected to the photodetector (PD), a continuously changing wide-band spectrum is displayed on the oscilloscope (OSC). Once the probe light is coupled to the microdisk through evanescent field coupling, light that satisfies the resonant conditions forms resonance modes within the microdisk, resulting in discrete dips in the transmission spectrum. By analyzing these resonant dips, we can gain a more intuitive understanding of the optical properties of the microdisk. Furthermore, the laser performance of the Nd:GGG microdisk is evaluated using the same experimental setup depicted in Figure 2, with switcher 1 switched to position A and switcher 2 switched to position A. In this case, an 808 nm semiconductor laser is employed as the pumping laser, which is also coupled into the microdisk via the tapered fiber using evanescent field coupling. The laser generated inside the microdisk is then coupled back into the tapered fiber and received by an optical spectrum analyzer (OSA) for analysis. To accurately determine the amount of pumping power coupled into the microdisk, we utilized a power meter (PM) to measure the power of the pumping laser both before and after coupling with the microdisk (switcher 2 set at B). This enabled us to obtain the precise value of the pumping power that was effectively coupled into the microdisk.

## 3 Results and discussion

**Figure 3:**
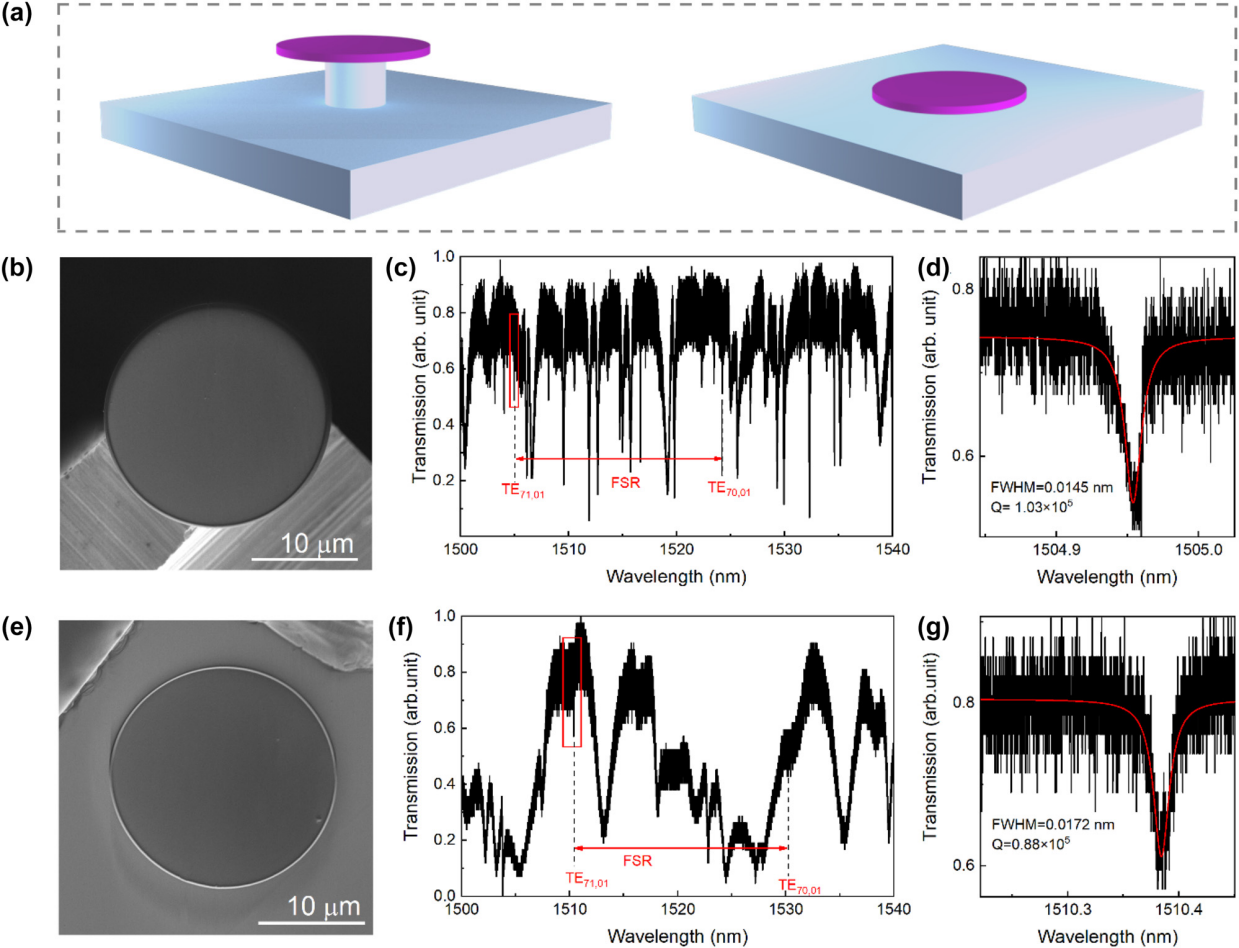
Characterization of Nd: GGG microdisks. (a) Schematic diagram of Nd:GGG microdisk on silica holder and on silica wafer; (b) SEM image of Nd:GGG microdisk on silica holder. Scale bar, 10 μm; (c) transmission spectrum of Nd:GGG microdisk on silica holder at 1500–1540 nm; (d) zoom-in image of the resonance mode marked by red box in (c), with FWHM of 0.0145 nm and *Q* factor of 1.03 × 10^5^; (e) SEM image of Nd:GGG microdisk on silica wafer. Scale bar, 10 μm; (f) transmission spectrum of Nd:GGG microdisk on silica wafer at 1500–1540 nm; (g) zoom-in image of the resonance mode marked by red box in (f), with FWHM of 0.0172 nm and *Q* factor of 0.88 × 10^5^.

As depicted in Figure 3a, the Nd:GGG microdisk, with a diameter of 20 μm, was transferred onto a silica holder and silica wafer, respectively. The SEM images of the holder-based and wafer-based microdisks are presented in Figure 3b–e, respectively. By utilizing the experimental setup illustrated in Figure 2, we measured their transmission spectrum. The transmission spectrum of the holder-based microdisk, spanning from 1500 to 1540 nm, is displayed in Figure 3c, wherein the free spectral range (FSR) between TE_71,01_ and TE_70,01_ is measured to be 19.3 nm. The zoomed-in plot of the resonance mode TE_71,01_, indicated by the red box, is depicted in Figure 3d. Employing Lorentz fitting (red line), we deduce a full-width at half-maximum (FWHM) of 0.0145 nm, corresponding to a loaded *Q* factor of 1.03 × 10^5^. Due to the larger thickness and higher refractive index of the Nd:GGG microdisk, the influence of surface roughness on the TE fundamental mode is relatively weak. Figure 3f shows the transmission spectrum of the wafer-based microdisk, exhibiting a higher FSR of 19.8 nm compared to the holder-based microdisk. This discrepancy is attributed to the redshift of TE_71,01_ of the wafer-based microdisk, as the TE_71,01_ mode has higher effective refractive index in wafer-based microdisk than the holder-based one due to the contact with silica wafer. Figure 3g illustrates the *Q* factor of the wafer-based microdisk to be 0.88 × 10^5^, slightly lower than that of the holder-based microdisk. We believe it is because the introduction of impurities between the microdisk and the wafer during the multiple transfer processes.

**Figure 4:**
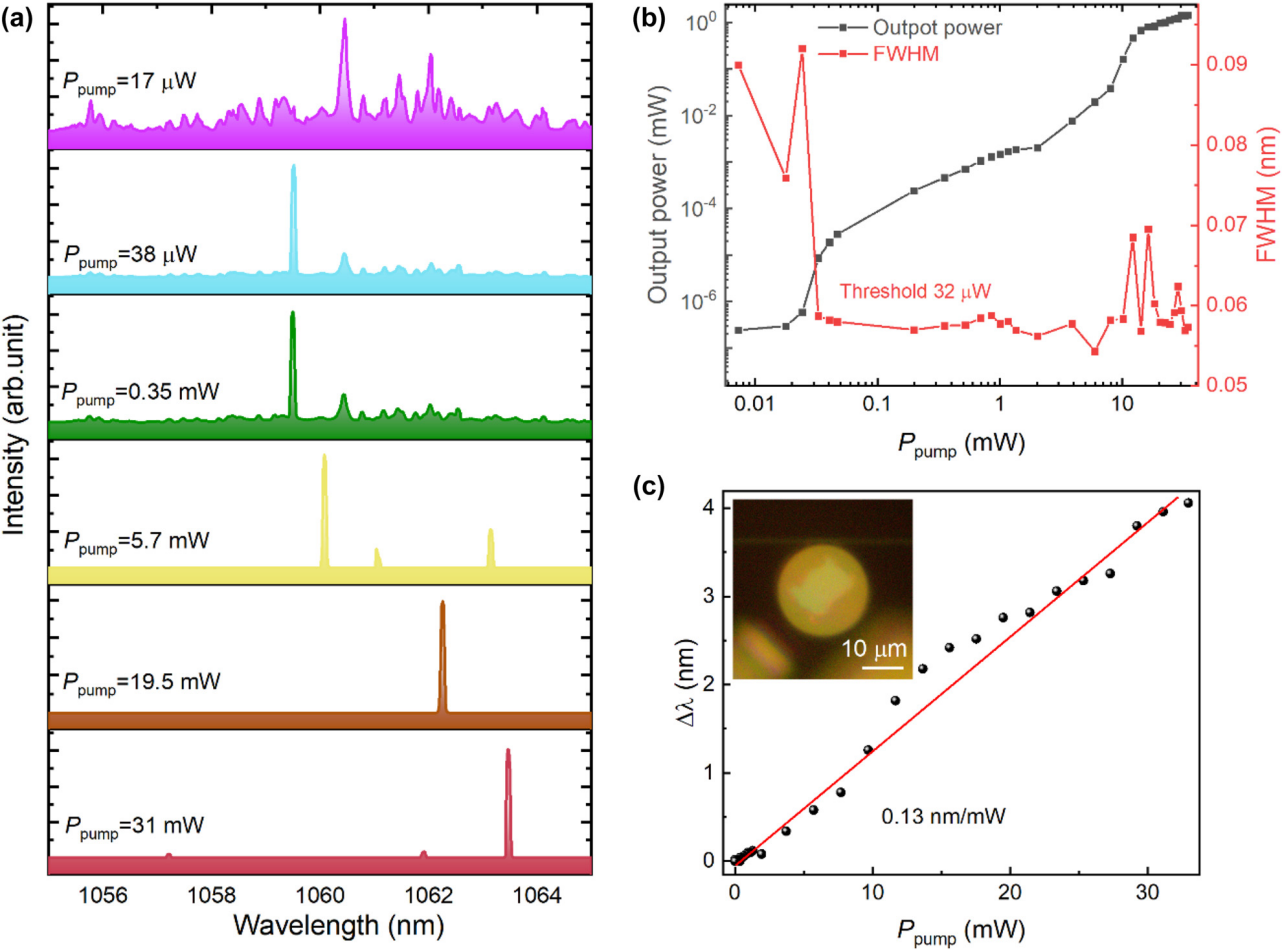
Laser operation of Nd:GGG microdisk on silica holder. (a) Emission spectra of Nd:GGG microdisk on silica holder with different *P*_pump_; (b) output power and FWHM of lasing mode with different *P*_pump_; (c) wavelength shift of lasing mode with different *P*_pump_. Illustration: optical image of Nd:GGG on silica holder coupled with tapered fiber. Scale bar, 10 μm.

Figure 4 illustrates the laser characteristics of the holder-based microdisk. As shown in Figure 4a, there are multiple resonance modes in the output spectrum, with the majority of the output energy concentrated around the 1059.52 nm mode. Figure 4b displays the evolution of output power and FWHM of the 1059.52 nm mode along with the pumping power. The FWHM here is obtained through Lorentzian fitting of the mode in emission spectra. With the increasing pumping power (coupled into the microdisk, denoted as *P*_pump_), the output power of the lasing mode gradually increases, with the FWHM fluctuating around 0.08 nm. When *P*_pump_ exceeds 32 μW, the output power of the lasing mode rapidly increases, while the FWHM decreases to around 0.06 nm, indicating the laser threshold is reached at *P*_pump_ = 32 μW. With further increase in *P*_pump_, the output power continuously increases and eventually reaches a maximum output power of 1.4 mW, and maximum optical conversion efficiency of 4.9 %. Figure 4a demonstrates the output spectra at different *P*_pump_. When *P*_pump_ is 17 μW (below the threshold), the energy is concentrated in the resonance mode at 1060.47 nm, while the resonance mode at 1059.52 nm is suppressed. When *P*_pump_ is 38 μW, the energy is concentrated in the mode at 1059.52 nm, and the FWHM of this mode decreases. With increasing *P*_pump_, the temperature of the laser increases accordingly, and the lasing mode exhibits a noticeable redshift (Figure 4a). The observed redshift (Δ*λ*) in the lasing mode is attributed to the optothermal effect and thermal expansion caused by the heat generated during laser operation, and can be described by the following formula:

(1) $$\Delta \lambda =\lambda_{0}\left(\frac{1}{n}\frac{dn}{dT}+\frac{1}{D}\frac{dD}{dT}\right)\Delta T,$$

where *λ*_0_ is the initial wavelength at the room temperature, *dn*/*dt* refers to the thermo-optic coefficient, (1/*D*)(*dD*/*dT*) designates the thermal expansion coefficient, and Δ*T* is defined as the change in temperature. The lasing mode approximately linearly drifts with the *P*_pump_ at the slope of 0.13 nm/mW, as shown in Figure 4c. When *P*_pump_ reaches 31 mW, Δ*λ* reaches 4.16 nm.

**Figure 5:**
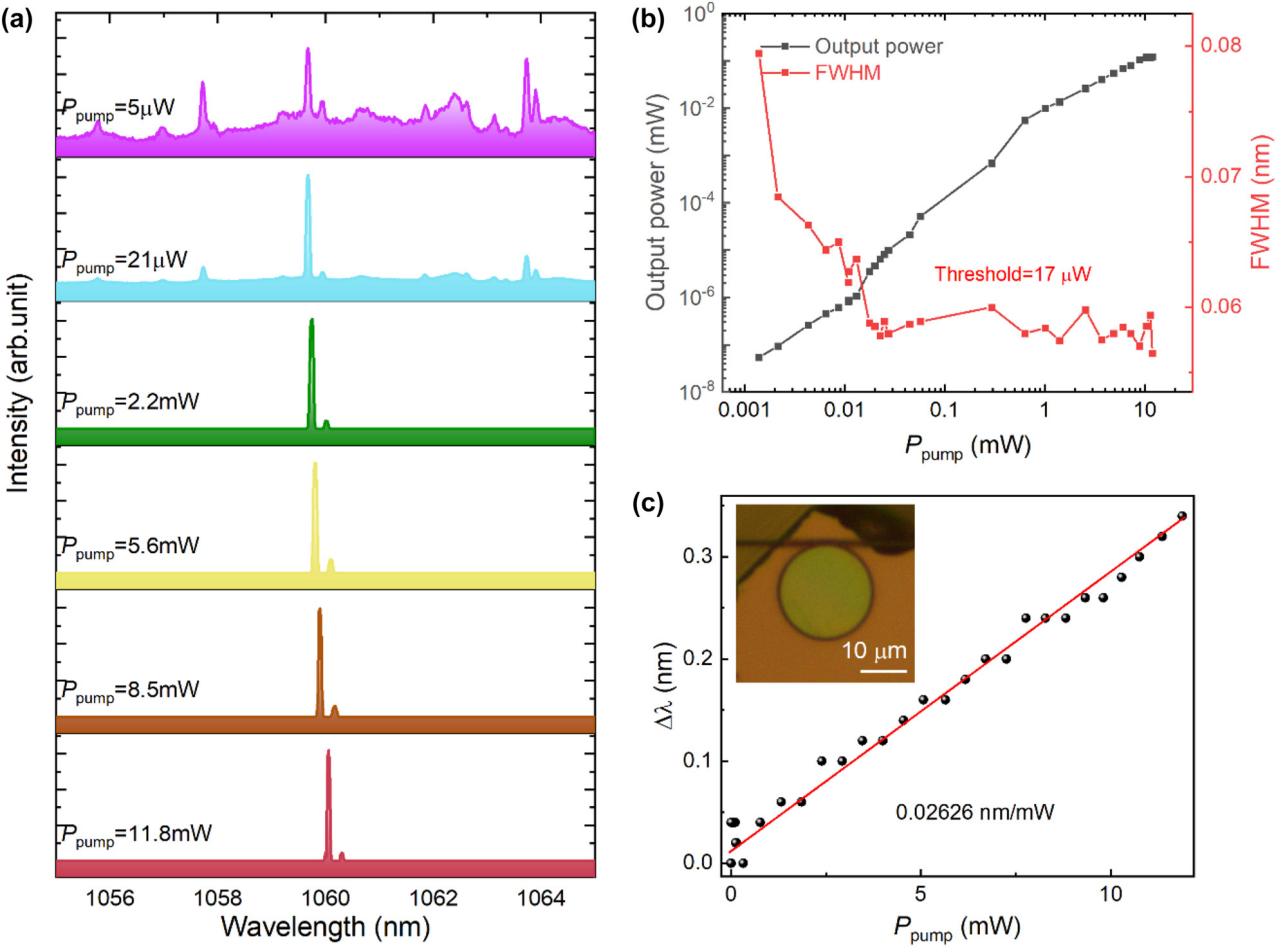
Laser operation of Nd:GGG microdisk on silica. (a) Emission spectra of Nd:GGG microdisk on silica wafer with different *P*_pump_; (b) output power and FWHM of lasing mode with different *P*_pump_; (c) wavelength shift of lasing mode with different *P*_pump_. Illustration: optical image of Nd:GGG on silica wafer coupled with tapered fiber. Scale bar, 10 μm.

The laser performance of the wafer-based microdisk is shown in Figure 5. As shown in Figure 5a, resonant modes are significantly reduced, with the energy primarily concentrated at the mode centered at 1059.68 nm. Figure 5b illustrates the dependence of output power on *P*_pump_. The output power increases with the increasing *P*_pump_, and there is a turning point at *P*_pump_ = 17 μW. With *P*_pump_ less than 17 μW, the FWHM of the resonance modes decreases as *P*_pump_ increases. When *P*_pump_ exceeds 17 μW, the slope of the output power becomes larger, and the FWHM stabilizes around 0.06 nm. This process indicates a laser threshold of 17 μW. For wafer-based microdisk, due to ineffective resonances for higher-order modes, the efficiency of pumping laser utilization decreases. Thus, the maximum output power is measured to be 120 μW with *P*_pump_ of 11.8 mW, and the maximum optical conversion efficiency of 1.2 %. Figure 5a presents the emission spectra under different *P*_pump_ in the range of 1055–1065 nm. When *P*_pump_ is below the threshold, the main energy of the emission spectrum is concentrated within the resonance mode at 1059.68 nm. When *P*_pump_ increases to 21 μW, the energy is further concentrated in the resonance mode at 1059.68 nm, which is accompanied by a decrease in FWHM. With further increase in *P*_pump_, a slight redshift of the lasing mode occurs. We analyzed the shift in center wavelength of the lasing mode versus different *P*_pump_ (Figure 5c) and discovered that the redshift of the lasing mode undergo a liner increase with the slope of 0.02626 nm/mW as *P*_pump_ increases.

As discuss from Figures 4 and 5, the threshold of the Nd:GGG microdisk on silica wafer is significantly lower than the threshold observed for microdisk which placed on silica holder. Generally, resonator with high *Q* factor exhibit less losses leading to lower laser threshold. However, the experimental results contradict the *Q* factor obtained in Figure 3. Combining the information provided by the output spectra in Figures 4a and 5a, it becomes apparent that the microdisk placed on silica wafer contain fewer resonance modes in the gain band of Nd:GGG than that in holder-based microdisk. Excessive resonant modes of the holder-based microdisk in the gain region lead to mode competition, which suppressing the laser performance. Therefore the microdisk on silica wafer exhibits a lower laser threshold. On the other hand, unlike the gradual reduction of FWHM in Figure 5b, the FWHM in Figure 4b experiences a steep decline, which highlights the presence of mode competition in the microdisk on silica holder. The lower optical conversion efficiency observed of the microdisk on silica wafer can be attributed to the non-uniform gain in Nd:GGG. As the microdisk on silica holder undergoes larger mode shifting, the laser mode moves to 1062 nm with high *P*_pump_. From Figure 1c, it is evident that the emission cross-section at 1062 nm is significantly higher than at 1059 nm. In addition, the difference between the two structures is also evident in the redshift of the lasing mode with respect to *P*_pump_. As mentioned above, under the same pumping conditions, the redshift of the microdisk on silica wafer is noticeably slower than that on the silica holder. We attribute this discrepancy of laser operation to the difference of thermal conductivity of air (∼0.0003 W cm^−1^ K^−1^) and silica (∼0.27 W cm^−1^ K^−1^). The wafer-based microdisk has better heat dissipation conditions, enabling more stable laser output.

## 4 Conclusions

In summary, we have utilized ion implantation to introduce defects into Nd:GGG crystal, creating a sacrificial layer which is subsequently etched away to obtain Nd:GGG crystalline thin films. Using FIB milling, these thin films are patterned into microdisk with the thickness of 1.8 μm and the diameter of 20 μm. To compare the impact of different substrates on laser performance, we situated the microdisk on a silica holder and a silica wafer, respectively. The microdisk placed on the silica holder exhibited a *Q* factor of 1.03 × 10^5^ in the C-band, whereas the microdisk situated on the silica wafer exhibited a slightly lower *Q* factor of 0.88 × 10^5^. The laser threshold for the Nd:GGG microdisk on silica holder is 37 μW, with a maximum output power of 1.4 mW and maximum optical conversion efficiency of 4.9 %. However, due to the low thermal conductivity of air, the heat generated during laser operation cannot be effectively dissipated, which resulted in a frequency shift with a slope of 0.13 nm/mW. Conversely, the microdisk directly placed on silica wafer has a laser threshold of 17 μW, the maximum optical conversion efficiency of 1.2 % and its stable laser operation is facilitated by the excellent thermal conductivity of silica, leading to a significantly reduced slope of 0.02626 nm/mW for the redshift of the lasing mode. The implementation of Nd:GGG microdisk lasers presents a novel option for on-chip high-power optical pumping microlaser. At the same time, the near-infrared microcavity laser demonstrated here exhibits potential applications in environmental monitoring and biomedical.
